# Analysis of sociodemographic and clinical factors associated with Lassa fever disease and mortality in Nigeria

**DOI:** 10.1371/journal.pgph.0000191

**Published:** 2022-08-26

**Authors:** Adebola T. Olayinka, Kelly Elimian, Oladipupo Ipadeola, Chioma Dan-Nwafor, Jack Gibson, Chinwe Ochu, Yuki Furuse, Akanimo Iniobong, Adejoke Akano, Lorna Enenche, Michael Onoja, Chukwuemeka Uzoho, Nkem Ugbogulu, Favour Makava, Chinedu Arinze, Geoffrey Namara, Esther Muwanguzi, Kamji Jan, Winifred Ukponu, Tochi Okwor, Chimezie Anueyiagu, Muhammad Saleh, Anthony Ahumibe, Chibuzo Eneh, Elsie Ilori, Nwando Mba, Chikwe Ihekweazu

**Affiliations:** 1 World Health Organisation, Abuja, FCT, Nigeria; 2 Nigeria Centre for Disease Control, Abuja, FCT, Nigeria; 3 Centers for Disease Prevention and Control, Abuja, Nigeria; 4 University of Nottingham, Nottingham, United Kingdom; 5 Georgetown University Centre for Global Health Practice and Impact, Abuja, Nigeria; Instituto Butantan, BRAZIL

## Abstract

Over past decades, there has been increasing geographical spread of Lassa fever (LF) cases across Nigeria and other countries in West Africa. This increase has been associated with significant morbidity and mortality despite increasing focus on the disease by both local and international scientists. Many of these studies on LF have been limited to few specialised centres in the country. This study was done to identify sociodemographic and clinical predictors of LF disease and related deaths across Nigeria. We analysed retrospective surveillance data on suspected LF cases collected during January-June 2018 and 2019. Multivariable logistic regression analyses were used to identify the factors independently associated with laboratory–confirmed LF diagnosis, and with LF–related deaths. There were confirmed 815 of 1991 suspected LF cases with complete records during this period. Of these, 724/815 confirmed cases had known clinical outcomes, of whom 100 died. LF confirmation was associated with presentation of gastrointestinal tract (aOR 3.47, 95% CI: 2.79–4.32), ear, nose and throat (aOR 2.73, 95% CI: 1.80–4.15), general systemic (aOR 2.12, 95% CI: 1.65–2.70) and chest/respiratory (aOR 1.71, 95% CI: 1.28–2.29) symptoms. Other factors were being male (aOR 1.32, 95% CI: 1.06–1.63), doing business/trading (aOR 2.16, 95% CI: 1.47–3.16) and farming (aOR 1.73, 95% CI: 1.12–2.68). Factors associated with LF mortality were a one-year increase in age (aOR 1.03, 95% CI: 1.01–1.04), bleeding (aOR 2.07, 95% CI: 1.07–4.00), and central nervous manifestations (aOR 5.02, 95% CI: 3.12–10.16). Diverse factors were associated with both LF disease and related death. A closer look at patterns of clinical variables would be helpful to support early detection and management of cases. The findings would also be useful for planning preparedness and response interventions against LF in the country and region.

## Introduction

Lassa fever (LF) is a viral hemorrhagic disease transmitted from infected rodents to humans or through human-to-human contacts [1]. Generally, animal-to-human transmission occurs following exposure to rodent excreta and secretions (urine and saliva) while human-to-human transmission occurs via contact with body fluids of an infected person and is related to poor infection control practices [1]. Historical data indicate that LF is endemic in West African countries of Nigeria, Sierra Leone, Liberia, Benin and Guinea, with sporadic cases occurring elsewhere in the region [2]. In West Africa, the disease is widespread, affecting an estimated two million persons and deaths of 5,000–10,000 persons annually [2]. Nigeria has reported the greatest number of confirmed LF cases in the region, accounting for 66% & 67% of all reported LF cases in 2018 & 2019 respectively [3]. Overall, there were 1,463 reported LF confirmed cases and 344 (23.5%) deaths in Nigeria during the 2018 and 2019 outbreaks, with 23 of the 36 states reporting confirmed cases [4].

The increase in number and geographical spread of reported LF cases across Nigeria has been attributed to improvement in the surveillance and laboratory system, which has enhanced the detection of cases compared to what obtained previously [5]. Nevertheless, there is paucity of evidence on factors associated with LF disease and deaths, for context-specific public health planning and interventions. From literature review, we found that available studies are mainly on description of epidemiologic and clinical features, on trends of LF and on nosocomial transmission of LF in health facilities [5–7]. Previous epidemiological studies which assessed factors associated with LF in Nigeria have usually been limited to specific localities and these haven’t provided comprehensive evidence of the national situation [8–10]. Our study aimed to address this gap by identifying the sociodemographic and clinical predictors of disease and death due to LF across many states in Nigeria.

## Method

### Study design and context

This was an analysis of retrospective LF surveillance data from all the 36 states in Nigeria, including the Federal Capital Territory (FCT), between January 2018-June 2018 and January 2019-June 2019 which covers the high transmission season for LF in Nigeria. Study population included all ages and both sexes across the 36 states plus the FCT of Nigeria. All cases were Nigerians and residents of the country as of the time the data was recorded. The provision of health care and disease surveillance services in Nigeria are the responsibility of state and federal governments involving primary, secondary and tertiary health facilities, with contribution from the private health sector [11]. This study is reported as per the “Strengthening the Reporting of Observational Studies in Epidemiology” (STROBE) guideline [S1 Checklist].

#### Data source

Surveillance, Outbreak Response Management and Analysis System (SORMAS) electronic health surveillance database, processed in a central server at Nigeria Centre for Disease Control (NCDC) in Abuja, Nigeria served as the data source for this study. SORMAS is currently the primary digital platform for implementing the Integrated Disease Surveillance and Response system in Nigeria [12]. Public health officers and epidemiologists across the country use SORMAS and in 2019 a platform for clinicians, nurses and laboratory staff was added. SORMAS is accessed either via mobile tablets or desktop computers.

The analysis plan for the study started with request for permission from the Director General of NCDC for access to SORMAS data on Lassa fever for years 2018 to 2019. A protocol was developed and submitted to the National Health Research and Ethics Committee of Nigeria for approval. The data which excluded identifiers was exported from SORMAS to Excel for analysis. The list of all epidemiologic variables and clinical variables available on the platform were generated and data were checked for completeness and to identify proportion of missing data across all variables. Variables with more than 10% missing data were excluded from analysis. Outcome and independent variables had been pre-defined.

### Data collection of clinical and socio-demographic variables

National guidelines on routine surveillance of suspected LF cases involves the collection of blood sample from all LF suspected cases in Ethylenediaminetetraacetic acid (EDTA) bottle either at inpatient or outpatient (including accident and emergency) departments of a health facility [13]. The sample is then safely transported in cold chain to one of the four LF reference laboratories in the country for confirmatory test by reverse transcriptase polymerase chain reaction (RT-PCR). This test targets the glycoprotein complex (GPC) gene using QIAGEN OneStep RT-PCR Kit reagents (Qiagen, no. 210210 or 210212). Testing (based on the national case definition for suspected cases) is indicated for any patient with fever (temperature ≥38°C) occurring in the last 21 days before presentation and with one or more signs and symptoms (vomiting, diarrhoea, sore throat, myalgia, generalised body weakness, abnormal bleeding or abdominal pain. Neonates were tested if the mother had Lassa fever, regardless of presence or absence of symptoms in the neonate. Collected blood samples were transported in cold chain to one of the four LF reference laboratories in Nigeria for confirmatory test by reverse transcriptase polymerase chain reaction (RT-PCR) [13]. LF confirmation in Nigeria is based on a positive RT-PCR test. All laboratory confirmed LF cases were managed in-line with existing treatment protocol which includes admission in isolation wards, supportive care and the administration of Ribavirin to all confirmed cases including pregnant females [14]. In addition to clinical diagnosis, patients’ socio-demographic information, signs and symptoms at presentation, laboratory findings, and clinical outcome (survived, died or discharged against medical recommendation) were recorded into the electronic version of the national case investigation form in SORMAS [S1 and S2 Files].

### Handling of missing data

Fully anonymised data on LF were retrieved from the SORMAS database within the Lassa Fever Technical Working Group at NCDC. Many of the variables including clinical had missing data. We adopted a complete-case approach to the analysis of socio-demographic variables (age group, sex and occupation/job category). We however excluded variables with more than 10% missing data (time to health seeking, self-report of travel outside of locality, <21 days prior to illness onset, and self-report of contact with rodents) from the analyses to minimise loss of power.

### Definition of key study variables

#### Outcome variables

The two outcome variables for this study were: (1) LF disease, defined as a suspected case that is subsequently laboratory confirmed (RT-PCR) either positive or negative for LASV, and (2) clinical outcome of a confirmed case as (alive or dead). Both variables were treated as binary.

#### Independent variables/covariates

Variables presumed to be potentially associated with both outcome variables were identified based on evidence in the literature and biological plausibility [5, 6, 9, 10, 15, 16]. Age in years, was based on self-report by patients/relatives. Occupation/Job category were grouped as follows: Unemployed/uncategorised, artisan/unskilled job, business/trading, agricultural workers/farmer, community leader, wage-earning job and health-related job. This was to explore the association between what people are engaged in and the risk of LF. A lot of these jobs however have loose definitions eg Business is assumed to involve higher capital investment than trading, but in the society, people use the terms interchangeably. Given the high unemployment rate in Nigeria (increasing from 18.8% in the third quarter of 2017 to 23.1% in the same quarter of 2018 in a population of over 180million people. [17] we assumed that individuals without a specific occupational category were most likely to be unemployed (e.g. housewife, students, pupils) during the study period. To minimise unstable estimates of effect from small sample numbers (<5), which was the case for most clinical variables, we decided to merge individual clinical signs or symptoms associated with LF into distinct syndromes based on a consensus reached by the study clinicians who were drawn from the LF treatment centres. The clinical signs and laboratory parameters presented were as documented in records. The composition of each clinical variable is summarised in Table 1 below.

**Table 1:** Clinical variables and their compositions.

Clinical variable	Composition
**General systemic symptoms†**	Maculopapular rash, anorexia/loss appetite, refusal to eat/drink, fatigue/malaise, headache, muscle/side pain, joint pain/arthritis, nausea or jaundice.
**Chest or respiratory symptoms**	Difficulty breathing, cough, or chest pain
**Bleeding symptoms**	Blood in urine/haematuria, bloody stool, black stool (meleana), coughing of blood, bleeding from nose/epistaxis, bleeding gum, bruising of the skin, bleeding from injection site, digested blood/coffee grounds in vomit, fresh/red blood in vomit/bleeding from the stomach, bleed from the eye or unusual vaginal bleeding
**Central nervous system symptoms**	Unconsciousness, confusion, convulsion
**Ophthalmic symptoms**	conjunctivitis/eye pain/sensitivity to light
**Ear, nose and throat (ENT) symptoms**	Sore throat/pharyngitis, acute hearing loss, or pharyngeal exudates.
**Gastrointestinal tract symptoms**	Dehydration, sunken eyes/fontanelle, diarrhoea, abdominal pain, or vomiting.
**Out of range laboratory parameters**	Hyperglycemia, hypoglycemia or thrombocytopenia.
**Other clinical signs**	Palpable liver, palpable spleen, fluid in lung cavity, sepsis, shock (systolic BP<90) or tremor.

†Fever was not considered as a potential component of general systemic symptoms being one of the defining symptoms for deciding Lassa fever diagnosis. As such, its assessment would not have made any difference, or biased the final estimates.

### Statistical analyses

Analyses were carried out in STATA version 13 (Stata Corp. LP, College Station, TX, United States of America), a p-value of <0.05 was considered statistically significant. Demographic and clinical characteristics of the study participants in relation to both outcome variables were described in terms of frequencies and percentages (%) for binary/categorical variables, and with mean and standard deviation (SD) for normally distributed continuous variables, unless indicated otherwise. To assess the association between individual covariate and outcome variables, we conducted univariable logistic regression analyses in turn for each outcome variable and presented the findings as unadjusted odds ratios (ORs) and 95% Confidence Intervals (95% CIs). This was then followed by multivariable analyses using a stepwise multiple logistic regression approach with all statistically significant covariates in the univariable model included in the multivariable model, and removed one at a time from the model based on their statistical significance (p-values from the LRT were used for categorical variables while the p-values from Wald’s test were used for binary and continuous variables) until all the covariates left in the model were statistically significant. Findings from the multivariable model were presented as adjusted ORs and 95% CIs.

### Ethics

The protocol for this study was reviewed and approval and waiver of consent given by the Nigeria National Health Research Ethics Committee (Approval Number: NHREC/01/01/2007-07/09/2020) The ethical principles on anonymity and confidentiality of records in the conduct of research activities were strictly adhered to throughout the conduct of this study.

## Results

### Socio-demographic and clinical characteristics of the study population

For the study, 1,991 cases of suspected LF had complete records of laboratory diagnosis for the 2 years in review, of which 815 (40.9%) were RT-PCR LF confirmed cases (Table 2). Of the confirmed LF cases, 724 persons had complete records of clinical outcome (alive or dead), of whom 100 died, giving a case fatality rate of 13.8%. Proportion of males with LF was (58.5%), death was 61.0% in males vs 39.0% in females. Highest proportion of LF cases was recorded among those aged 25–44 years (41.8%) and a similar trend was recorded for deaths. Regarding clinical variables, general systemic symptoms (96.1%) were the most common signs and symptoms recorded among LF patients, followed by gastrointestinal tract (65.3%) and chest/respiratory (32.2%) symptoms. Similar pattern was also recorded among those who died from LF. Table 3 provides breakdown of the clinical manifestations in the database and laboratory parameters recorded by clinicians. These could not be compared against those who tested negative as many LF negative patients are not admitted nor their clinical data recorded in SORMAS.

**Table 2:** Baseline characteristics of the study population in relation to Lassa fever confirmation and death.

Lassa fever confirmation Clinical outcome				
Characteristic	PCR positive [n = 815]	All PCR-tested cases [n = 1,991]	Dead [n = 100]	All LF cases with clinical outcome [n = 724]
	n (%)	n (%)	n (%)	n (%)
**Epidemic year**				
Jan.-Jun. 2018	224 (27.48)	298 (14.97)	22 (22.00)	196 (27.07)
Jan.-Jun. 2019	591 (72.52)	1,693 (85.03)	78 (78.00)	528 (72.93)
**Sexδ**				
Female	337 (41.40)	908 (45.74)	39 (39.00)	304 (41.99)
Male	477 (58.60)	1,077 (54.26)	61 (61.00)	420 (58.01)
**Mean age (SD), years**	32.04 (17.33)	29.9 (19.2)	39.06 (18.81)	32.30 (17.58)
**Age groups, year§**				
0–4	37 (4.73)	137 (6.88)	3 (3.13)	35 (4.99)
5–14	91 (11.62)	270 (13.56)	5 (5.21)	79 (11.27)
15–24	146 (18.65)	395 (19.84)	9 (9.38)	131 (18.69)
25–34	165 (21.07)	403 (20.24)	19 (19.79)	145 (20.68)
35–44	175 (22.35)	338 (16.98)	25 (26.04)	153 (21.83)
45–54	87 (11.11)	185 (9.29)	17 (17.71)	80 (11.41)
55–64	41 (5.24)	101 (5.07)	6 (6.25)	40 (5.71)
≥65	41 (5.24)	109 (5.47)	12 (12.50)	38 (5.42)
**Occupation/Job category**				
Unemployed/uncategorised	521 (63.93)	1,472 (73.93)	55 (55.00)	454 (62.71)
Artisan/unskilled job	44 (5.40)	72 (3.62)	10 (10.00)	35 (4.83)
Business/trading	91 (11.17)	164 (8.24)	12 (12.00)	81 (11.19)
Agricultural workers/farmers	70 (8.59)	126 (6.33)	15 (15.00)	68 (9.39)
Community leaders	8 (0.98)	11 (0.55)	1 (1.00)	7 (0.97)
Wage earning job (Civil servants, privately employed)	53 (6.50))	93 (4.67)	4 (4.00)	52 (7.18)
Health-related occupation	28 (3.44)	53 (2.66)	3 (3.00)	27 (3.73)
**Geopolitical zone of residenceǂ**				
North-central	56 (6.87)	156 (7.84)	13 (13.00)	46 (6.35)
South-south	348 (42.70)	962 (48.32)	23 (23.00)	300 (41.44)
South-east	87 (10.67)	170 (8.54)	23 (23.00)	78 (10.77)
South-west	219 (26.87)	484 (24.31)	30 (30.00)	211 (29.14)
North-west	13 (1.60)	48 (2.41)	3 (3.00)	8 (1.10)
North-east	92 (11.29)	171 (8.59)	8 (8.00)	81 (11.19)
**Setting**				
Rural	464 (56.93)	1,202 (60.37)	54 (54.00)	404 (55.80)
Semi-urban	39 (4.79)	93 (4.67)	6 (6.00)	33 (4.56)
Urban	312 (38.28)	696 (34.96)	40 (40.00)	287 (39.64)
**Initial facility of presentation**				
Primary	66 (8.10)	193 (9.69)	14 (14.00)	61 (8.43)
Secondary	19 (2.33)	42 (2.11)	4 (4.00)	19 (2.62)
Tertiary	636 (78.04)	1,543 (77.50)	77 (77.00)	578 (79.83)
Private	13 (1.60)	17 (0.85)	1 (1.00)	13 (1.80)
Unspecified	81 (9.94)	196 (9.84)	4 (4.00)	53 (7.32)
**Transferred cases**				
No	702 (86.13)	1,718 (86.29)	87 (87.00)	638 (88.12)
Yes	34 (4.17)	80 (4.02)	8 (8.00)	33 (4.56)
Unknown	79 (9.69)	193 (9.69)	5 (5.00)	53 (7.32)
**General systemic symptoms**				
No	164 (20.12)	640 (32.14)	9 (9.00)	139 (19.20)
Yes	651 (79.88)	1,351 (67.86)	91 (91.00)	585 (80.80)
**Chest/respiratory symptoms**				
No	553 (67.85)	1,601 (80.41)	52 (52.00)	485 (66.99)
Yes	262 (32.15)	390 (19.59)	48 (48.00)	239 (33.01)
**Bleeding symptoms**				
No	712 (87.36)	1,814 (91.11)	71 (71.00)	633 (87.43)
Yes	103 (12.64)	177 (8.89)	29 (29.00)	91 (12.57)
**Central nervous system symptoms**				
No	726 (89.08)	1,859 (93.37)	63 (63.00)	639 (88.26)
Yes	89 (10.92)	132 (6.63)	37 (37.00)	85 (11.74)
**Ophthalmic symptoms**				
No	799 (98.04)	1968 (98.84)	24 (25.00)	204 (28.18)
Yes	16 (1.96)	23 (1.16)	76 (76.00)	520 (71.82)
**Ear, nose & throat (ENT) symptoms**				
No	680 (83.44)	1,815 (91.16)	72 (72.00)	608 (83.98)
Yes	135 (16.56)	176 (8.84)	28 (28.00)	116 (16.02)
**Gastrointestinal tract symptoms**				
No	283 (34.72)	1,148 (57.66)	26 (26.00)	251 (34.67)
Yes	532 (65.28)	843 (42.34)	74 (74.00)	473 (65.33)
**Out of range lab parameters**				
No	803 (98.53)	1,975 (99.20)	98 (98.00)	713 (98.48)
Yes	12 (1.47)	16 (0.80)	2 (2.00)	11 (1.52)
**Other clinical signs**				
**No**	776 (95.21)	1,925 (96.69)	86 (86.00)	689 (95.17)
**Yes**	39 (4.79)	66 (3.31)	14 (14.00)	35 (4.83)

**Table 3:** Distribution of out-of-range laboratory parameters and other clinical signs among study participants.

Description of variable	Freq (%)
**Out of range Laboratory parameters**	
Hyperglycemia	
No	6,049 (99.93)
Yes	4 (0.07)
Hypoglycemia	
No	6,049 (99.93)
Yes	4 (0.07)
Thrombocytopenia	
No	6,034 (99.69)
Yes	19 (0.31)
**Other clinical signs**	
Palpable liver	
No	6,026 (99.55)
Yes	27 (0.45)
Palpable spleen	
No	6,043 (99.83)
Yes	10 (0.17)
Fluid in lung cavity	
No	6,045 (99.87)
Yes	8 (0.13)
Sepsis	
No	6,008 (99.26)
Yes	45 (0.74)
Shock (systolic BP<90mmHg)	
No	6,020 (99.45)
Yes	33 (0.55)
Tremor	
No	6,046 (99.88)
Yes	7 (0.12)

### Factors associated with Lassa fever and death

Odds of LF disease were higher in males than in females [OR 1.35, 95% CI: 1.12–1.61]. When compared with those aged 0–4 years, the odds of LF increased with higher age groups, particularly among those aged 35–54 years and 45–54 years, whose odds of contracting LF increased almost three and two-fold, respectively. Regarding clinical variables, the odds of LF was about six times [OR 5.5, 95% CI: 3.83–7.90; p<0.001] and five times [OR 5.2, 95% CI: 4.31–6.35; p<0.001] higher among persons who presented with ENT and gastrointestinal tract symptoms, respectively, than in those without these symptoms. (Table 4).

**Table 4:** Logistic regression: Socio-demographic & clinical predictors of Lassa fever.

Characteristic	Unadjusted model OR (95% CI)	LRT p-value	Adjusted model OR (95% CI)	LRT p-value
**Epidemic year (n = 1,991)**				
Jan.-Jun. 2018	1.00	<0.001†	1.00	<0.001†
Jan.-Jun. 2019	0.18 (0.13–0.23)		0.22 (0.16–0.30)	
**Sex (n = 1,985)**				
Female	1.00	0.001†	1.00	0.011†
Male	1.35 (1.12–1.61)		1.32 (1.06–1.63)	
**Age group, year (n = 1,938)**				
0–4	1.00	<0.0001	1.00	0.3786
5–14	1.37 (0.87–2.16)		1.00 (0.60–1.65)	
15–24	1.58 (1.03–2.43)		1.09 (0.67–1.77)	
25–34	1.87 (1.22–2.87)		1.00 (0.60–1.63)	
35–44	2.90 (1.88–4.48)		1.50 (0.90–2.50)	
45–54	2.40 (1.49–3.86)		1.21 (0.69–2.12)	
55–64	1.85 (1.07–3.20)		0.90 (0.47–1.73)	
≥65	1.63 (0.95–2.80)		1.11 (0.59–2.10)	
**Occupation/Job category (n = 1,991)**				
Unemployed/uncategorised	1.00	<0.0001	1.00	0.0002
Artisan/unskilled job	2.87 (1.76–4.66)		1.74 (0.98–3.08)	
Business/trading	2.28 (1.64–3.15)		2.16 (1.47–3.16)	
Agricultural workers/farmer	2.28 (1.58–3.30)		1.73 (1.12–2.68)	
Community leader	4.87 (1.29–18.43)		3.82 (0.85–17.24)	
Wage-earning job	2.42 (1.58–3.70)		1.19 (0.72–1.97)	
Health-related job	2.04 (1.18–3.54)		1.88 (0.99–3.56)	
**Geopolitical zone**ǂ **(n = 1,991)**				
North-central	1.00	<0.0001	1.00	<0.0001
South-south	1.01 (0.71–1.44)		1.50 (0.97–2.32)	
South-east	1.87 (1.20–2.92)		1.39 (0.81–2.40)	
South-west	1.48 (1.02–2.14)		2.86 (1.81–4.53)	
North-west	0.66 (0.32–1.36)		1.93 (0.82–4.53)	
North-east	2.08 (1.33–3.24)		3.60 (2.08–6.26)	
**Setting (n = 1,991)**				
Rural	1.00	0.0289	1.00	0.6403
Semi-urban	1.15 (0.75–1.76)		1.17 (0.69–1.98)	
Urban	1.30 (1.07–1.56)		0.89 (0.63–1.25)	
**General systemic symptoms (n = 1,991)**				
No	1.00	<0.001†	1.00	<0.001†
Yes	2.70 (2.20–3.32)		2.12 (1.65–2.70)	
**Chest/respiratory symptoms (n = 1,991)**				
No	1.00	<0.001†	1.00	<0.001†
Yes	3.88 (3.07–4.91)		1.71 (1.28–2.29)	
**Bleeding symptoms (n = 1,991)**				
No	1.00	<0.001†	1.00	0.079†
Yes	2.15 (1.58–2.95)		0.68 (0.45–1.04)	
**Central nervous system symptoms (n = 1,991)**				
No	1.00	<0.001†	1.00	0.302†
Yes	3.03 (2.12–4.20)		1.25 (0.72–2.01)	
**Ophthalmic symptoms (n = 1,991)**				
No	1.00	0.012	1.00	0.325
Yes	1.81 (1.43–2.78)		1.364 (0.92–3.41)	
**Ear, nose & throat symptoms (n = 1,991)**				
No	1.00	<0.001†	1.00	<0.001†
Yes	5.50 (3.83–7.90)		2.73 (1.80–4.15)	
**Gastrointestinal tract symptoms (n = 1,991)**				
No	1.00	<0.001†	1.00	<0.001†
Yes	5.23 (4.31–6.35)		3.47 (2.79–4.32)	
**Out of range lab parameters (n = 1,991)**				
No	1.00	0.011†	1.00	0.583†
Yes	4.38 (1.41–13.62)		1.44 (0.40–5.26)	
**Other clinical signs (n = 1,991)**				
No	1.00	0.003†	1.00	0.122†
Yes	2.14 (1.30–3.52)		0.59 (0.30–1.15)	

OR = odds ratio; CI = Confidence Interval

LRT = Likelihood Ratio Test

† Wald’s p-value

Significant results are in bold texts

ǂ Substantial data missing from high disease burden areas so results need to be interpreted with caution

In the adjusted model, variables positively associated with LF disease were the male sex [adjusted OR 1.32, 95% CI: 1.06–1.63; p = 0.011], occupation in business /trading [adjusted OR 2.16, 95% CI: 1.47–3.16) and farming/agricultural work [adjusted OR 1.73, 95% CI: 1.12–2.68] as well as clinical presentation at diagnosis with general systemic symptoms [adjusted OR 2.12, 95% CI: 1.65–2.70; p<0.001], chest/respiratory [adjusted OR 1.71, 95% CI: 1.28–2.29; p<0.001), ENT [adjusted OR 2.73, 95% CI: 1.80–4.15] and gastrointestinal tract [adjusted OR 3.47, 95% CI: 2.79–4.32] symptoms.

Age and symptoms related to bleeding and central nervous system (CNS) symptoms were significantly associated with death from LF (Table 5). Specifically, a one-year increase in age from age 0–4 years was associated with a 3% increase in the chance of death from LF [adjusted OR 1.03, 95% CI: 1.01–1.04; p<0.001]. Presentation with bleeding and CNS symptoms were, respectively, associated with about two-fold [adjusted OR 2.07, 95% CI: 1.07–4.00; p = 0.030] and five-fold [adjusted OR 5.02, 95% CI: 3.12–10.16; p<0.001] increase in the odds of death from LF disease. Presentation with general systemic symptoms was however not significantly associated with death in the adjusted model [adjusted OR 1.42, 95% CI: 0.67–3.04; p = 0.361].

**Table 5:** Logistic regression results of socio-demographic and clinical factors associated with death among Lassa fever patients (N = 724).

Characteristic	Unadjusted model OR (95% CI)	LRT p-value	Adjusted model OR (95% CI)	LRT p-value
**Socio-demographic variables**				
**Epidemic year**				
Jan.-Jun. 2018	1.00	0.220†		
Jan.-Jun. 2019	1.37 (0.83–2.27)			
**Sex**				
Female	1.00	0.514†		
Male	1.15 (0.75–1.78)			
**Age, year**	1.02 (1.01–1.04)	<0.001†	1.03 (1.01–1.04)	<0.001†
**Occupation/Job category**				
Unemployed/uncategorised	1.00	0.0662		
Artisan/unskilled job	2.90 (1.32–6.37)			
Business/trading	1.26 (0.64–2.48)			
Agricultural worker/farmer	2.05 (1.08–3.89)			
Community leader	1.21 (0.14–10.23)			
Wage = earning job	0.60 (0.21–1.74)			
Health-related job	0.91 (0.26–3.11)			
**Geopolitical zoneǂ**				
North-central	1.00	<0.0001	1.00	0.0040
South-south	0.21 (0.09–0.46)		0.31 (0.12–0.77)	
South-east	1.06 (0.47–2.38)		0.85 (0.33–2.21)	
South-west	0.42 (0.19–0.89)		0.54 (0.22–1.33)	
North-west	1.52 (0.32–7.31)		1.03 (0.16–6.77)	
North-east	0.28 (0.11–0.74)		0.20 (0.07–0.60)	
**Setting**				
Rural	1.00	0.7555		
Semi-urban	1.44 (0.57–3.65)			
Urban	1.05 (0.68–1.63)			
**Place of presentation**				
Primary	1.00	0.1329		
Secondary	0.90 (0.26–3.14)			
Tertiary	0.52 (0.27–0.98)			
Private	0.28 (0.03–2.34)			
Unspecified	0.27 (0.08–0.90)			
**Transferred cases**				
No	1.00	0.1727		
Yes	2.03 (0.89–4.64)			
Unknown	0.66 (0.26–1.70)			
**Time to health seeking, days**				
0–5	1.00	0.5632		
>5	1.34 (0.76–2.37)			
Unknown	1.15 (0.63–2.10)			
**Clinical variables**				
**General systemic symptoms**				
No	1.00	0.007	1.00	0.361
Yes	2.66 (1.31–5.42)		1.42 (0.67–3.04)	
**Chest/respiratory symptoms**				
No	1.00	0.001†	1.00	0.854†
Yes	2.09 (1.36–3.21)		0.95 (0.54–1.67)	
**Bleeding symptoms**				
No	1.00	<0.001†	1.00	0.030†
Yes	3.70 (2.23–6.14)		2.07 (1.07–4.00)	
**Central nervous system symptoms**				
No	1.00	<0.001†	1.00	<0.001†
Yes	6.84 (3.82–14.22)		5.02 (3.12–10.16)	
**Ophthalmic symptoms**				
No	1.00	0.524		
Yes	1.34 (0.76–3.67)			
**Ear, nose & throat symptoms**				
No	1.00	0.001†	1.00	0.661†
Yes	2.37 (1.45–3.87)		1.15 (0.61–2.17)	
**Gastrointestinal tract symptoms**				
No	1.00	0.051†		
Yes	1.60 (0.99–2.58)			
**Out of range lab parameters**				
No	1.00	0.673†		
Yes	1.40 (0.30–6.55)			
**Other clinical signs**				
No	1.00	<0.001†	1.00	0.341†
Yes	4.67 (2.30–9.54)		1.58 (0.62–4.07)	

OR = odds ratio; CI = Confidence Interval

LRT = Likelihood ratio test

† = Wald’s p-value.

ǂ Substantial data missing from high disease burden regions so results interpreted with caution

## Discussion

From our study we have identified factors associated with both LF disease and LF-associated death in Nigeria. Summarily, sex, occupation, and certain clinical variables were significantly and independently associated with LF disease during the period under study. While age, presentation with bleeding and CNS symptoms were independently, significantly associated with death from LF.

We quantified the risk of LF disease with respect to various occupational groups or types of jobs. Persons engaged in trading or business and farming were more at risk of LF disease when compared with those without an employment (e.g. housewife, student, job applicant among others). High number of LF cases have been recorded among traders in Abakaliki in south-eastern Nigeria [18]. Agricultural workers in Nigeria, many of whom are into subsistence farming, are known to dry farm produce in open spaces, such as along road sideways [19], thereby potentially predisposing themselves to consumption of food items contaminated by rodent reservoirs. Mitigating the risk of LF among these populations would require development and dissemination of specific risk communication messages, especially via radio and television jingles. This medium has been identified as the preferred information platform by many groups [19]. It is also worth noting that the odds of LF disease among health workers during the study period was about two-fold, though was not statistically significant and health workers were not many among our study participants. Historically, nosocomial transmission of LF, with high case fatality rates among health workers of all cadre, is commonplace in Nigeria, as evidenced by previous studies in Plateau, Nassarawa, Borno [20] as well as Edo [21] Ebonyi [9, 18] states and across the entire country [2, 7]. This trend has been attributed to low index of suspicion of LF by health service providers, poor infection control practices and poor diagnostic capacity [18]. Prior to 2018, the availability and implementation of infection prevention and control (IPC) measures against LF in health facilities was sub-optimal in Nigeria. [21] including in tertiary health facilities [5]. However, with recent investments in IPC training and implementation of measures across the country, the IPC culture in health facilities has improved.

In the study by McCormick *et al* [15], a combination of fever, pharyngitis, retrosternal pain, and proteinuria were significantly associated with LF disease. these are similar to the composition of general systemic as well as ear, nose and throat symptoms in our study, which have also been found to be associated with LF disease in Nigeria [18]. This is also similar to other studies which found gastrointestinal tract, general systemic, and ENT clinical manifestations to be associated with LF [21]. Bleeding and CNS symptoms found to be associated with LF disease in previous studies [22], we found to be predictors of mortality in our study. Our findings on factors associated with death from LF infection are similar to existing evidence among 291 LF patients between 2011 and 2015 [23] where elevated serum concentrations of creatinine, aspartate aminotransferase, or potassium as indicative of poor prognosis, and acute kidney injury and CNS manifestations were identified as significant factors associated with death. Despite the lack of detailed laboratory variables to facilitate a direct comparison with these findings by Okokhere *et al*. [23] we found CNS-related symptoms to be significantly associated with death. While CNS manifestations of LF are believed to be involved in the later stages of the illness and characteristic of fatal cases [22], the pathogenesis for this mechanism of action is poorly understood and calls for further studies [16]. This is at variance with study of McCormick *et al*. who found the combination of fever, sore throat, and vomiting to be associated with death from LF [15]. The clinical signs and symptoms identified to be significantly associated with LF and death in the current study could be useful to frontline health workers especially in areas with limited diagnostic capacity [21], there is a need to further explore, using Nigerian data, the diagnostic capacity of common LF signs and symptoms in comparison with RT-PCR. This becomes even more imperative as the commonly used clinical algorithm for screening patients with suspected LF in Nigeria is based on positive predictive values that are prevalence-dependent on long time data from rural Sierra Leone [15].

LF occurs across all age groups and in both males and females, with varying degree of risks [8]. In the present study, however, age was not significantly associated with LF disease. This is in contrast with findings from other studies [15, 18], although different age group categories were adopted. We however found that a one-year increase in age increased the odds of death from LF infection by 3%. Similar trends have been reported [7, 23], albeit age was treated as a categorical variable in these studies. It is known that as one gets older, there is an increase in the occurrence of comorbidities and diminishing ability to elicit an adaptive immune response to pathogens, and can succumb to adverse clinical outcomes, such as death [20]. Being a male increased the risk of LF disease by 32% compared to being a female in the present study. This finding may be explained by socio-behavioural activities among males in a Nigerian setting. Traditionally, men are more likely to be engaged in risky occupational activities including hunting of animals [19], including rodents, for food or income; and other agricultural work, hence the higher tendency to be exposed to LF infection—this hypothesis is supported by the higher risk of LF among agricultural workers or farmers in this study. This finding is however not consistent with a previous study in Nigeria where females were at greater risk of LF than their male counterparts [9]. Prevailing cultural practices in Nigeria tend to place greater burden of caring for the sick on women, thereby predisposing them to risk of LF infection when home care is provided to a LF patient [24, 25]. Given the divergent evidence, a follow up study aimed at shedding more light on the dynamics of LF transmission with respect to age and gender and on detailed clinical trend is recommended.

### Study strengths and limitations

This study attempts to systematically identify sociodemographic and clinical factors associated with LF and death using a national dataset in Nigeria, that includes data not limited to specialised LF treatment centres but includes data from many health facilities that have had to manage LF cases thus, filling an important research gap, and providing robust evidence for national public health planning and intervention design. Furthermore, interference by differential diagnosis of other endemic febrile illnesses in Nigeria, such as yellow fever and malaria, are not an issue in the current study as LF diagnosis was confirmed by RT-PCR. We also made deliberate efforts to minimise the occurrence of follow-up bias by excluding cases without at least one clinical record from analysis. Majority of the managing health facilities did not have supportive laboratory structures, nor did they have facilities for dialysis or ICU care. So these clinical variables were missing from the analysis. However, in clinical practice. clinicians and frontline health workers, use symptom assessment to classify patients and take clinical decisions, so we analysed symptoms at presentation.

Limitations include analysis of data specific to only the high LF seasonal periods of both years and substantial missing data on some variables known to increase the risk of LF (e.g. contact with rodents) which was dropped from the analysis in order to minimise selection bias However, seasonality is known to be associated with LF in West African endemic countries [2, 3, 7] with higher odds of LF case notification during this period. The clinical platform for SORMAS was only deployed in 2019 which meant that available clinical and lab details were limited. We could therefore not explore the effects of these variables on LF infection given substantial missing data among the analysed dataset. These limitations however do not apply to our estimated associations with death.

### Generalisability of findings

Findings from this study have the potential to be generalisable in Nigeria as the analysed data were from all the states across Nigeria, including the FCT. However, the analysed data were hospital-based, meaning that cases which tested negative for LF and were not admitted could not be assessed further.

## Conclusion

The identified predictors of Lassa fever disease were being male, trading, agricultural occupation and presence of fever with other respiratory, ENT and gastrointestinal generalised systemic symptoms. This tallies with the current suspected case definition for LF, while bleeding and CNS symptoms were associated with death. To identify more specific clinical symptoms and signs that could serve as indicators for LF, there will be need for more detailed data from both those who test positive and those who test negative. The identified variables as predictors of LF disease are related to sociodemographic, health systems-related, environmental, and clinical factors. This re-emphasises the importance of a one-health approach to addressing LF, while also providing context-specific evidence for improved Lassa fever outbreak preparedness and response as well as pointers to its clinical case management in Nigeria and possibly other West African countries.

## Supporting information

S1 Checklist STROBE statement—checklist of items that should be included in reports of observational studies. (DOC)

S1 File Lassa fever case investigation form. (PDF)

S2 File National Lassa fever case management form. (PDF)
