# Supplementary material for: Analysis of sociodemographic and clinical factors associated with Lassa fever disease and mortality in Nigeria
Source: PLOS Glob Public Health. 2022 Aug 26;2(8):e0000191. doi: 10.1371/journal.pgph.0000191 (PMC10022364; doi:10.1371/journal.pgph.0000191)
Supplement: S1 File — (PDF) [file pgph.0000191.s002.pdf]

LASSA FEVER CASE INVESTIGATION FORM (LF-CIF Version 3, 2019)

Surname:____

Other Names: ____

Epid-Number: NIE -STA-LGA-YR-000 ____

Section 1 - Reporting Health Facility		Section 2 – Case Residential Address. Where does the case live?			
1	**State:**	7	**State:**		
2	**LGA:**	8	**LGA:**		
3	**Ward:**	9	**Ward:**		
4	**Name of Health Facility:**	10	**Settlement/Village/Town:**		
5	**Date Seen at Health Facility (DD-MM-YYYY):**	11	**Street Number and Name:**		
6	**Date of Onset of Disease (DD-MM-YYYY):**		**GPS Code:**	**Longitude**	
				**Latitude**	

Section 3 - Patient Information					
12	**Outpatient** [ ] **Inpatient** [ ]		16	**Educational Level**	
13	**Sex:** Male [ ] Female [ ]<br>If female pregnant? Yes [ ] No [ ]			[ ] No Formal Education	
14	**Date of Birth (DD-MM-YYYY):**			[ ] Primary	
	Years (Adult/children)			[ ] Secondary	
	Months (- Infants)			[ ] Tertiary	
	Days (Neonate)				
15	**Age (as at last birthday):**		19	**Occupation**	
17	**Phone Number**:			*(Farmer, Butcher, Hunter/trader of game meat, Miner, Religious leader, Housewife, Pupil/Student, Trader Artisan: Transporter; type of transport, Civil Servant; Health care worker; Traditional/spiritual healer, Unemployed, Specify Others)*	
18	**Owner of Phone Number**:				
20	**Present Condition**				
	Alive	[ ] At home [ ] On admission	**21. Name of treatment Centre:**		
	Dead	[ ] Died at Home [ ] Died on arrival at a hospital or treatment centre			
22	**Date of Death (DD-MM-YYYY):**				
23	**Mode of Burial**				
	Safe	[ ] Use of PPEs for burial, State and LGA team involved Corpse not exposed after putting in body bag			
	Unsafe	[ ] Body not put in a body bag			

Section 4 - Symptoms *(Please provide an answer for **all** symptoms)*									
24. Symptoms		Yes	No	Unknown	24. Symptoms		Yes	No	Unknown
A	Fever				N	Difficulty swallowing/ Sore throat			
B	Nausea/Vomiting				O	Jaundice (yellow eyes/gums/skin)			
C	Diarrhoea				P	Conjunctivitis (red eyes)			
D	Intense fatigue /general weakness				Q	Pain behind eyes/sensitive to light			
E	Loss of appetite				R	Hiccups			
F	Abdominal pain				S	Skin rash			
G	Chest pain				T	Coma/unconscious			
H	Muscle pain				U	Confused or disoriented			
I	Joint pain				V	Chills			
j	Oedema (body swelling)					Unexplained bleeding from any site			
K	Headache					1) Bleeding of the gums			
L	Cough					2) Bleeding from injection site			
M	Difficulty in breathing					3) Nose bleeding (epistaxis)			
	Others:					4) Bleeding from ear			
						5) Bleeding from vagina/Foetal-loss			
						6) Others specify ____________			

	Section 5: Source of infection<br>Please check the appropriate box for each question	Yes	No	Unknown
25. Is the case linked to a contact tracing list?<br>If yes, Provide Epid Number of the source case________________________________				
1)	In the three weeks **before** you became unwell, did you have contact with a **confirmed** Lassa fever case?			
	a) If yes, who?			
	b) If yes, approximately when in the last 3weeks			
2)	In the three weeks **before** you became unwell, did you have contact with an **unwell person**?			
	a) If yes, who?			
	b) If yes, approximately when in the last 3 weeks			
3)	Did you travel away from home in the 3 weeks **before** you became ill?			
	If yes, where and when did you travel (location and date) ________________? Duration of Trip: From DD/MM/YYYY to DD/MM/YYYY			
4)	The following questions are about any contact with rodents:			
	a) In the 3 weeks **before** you became ill did you **hunt** any rodents?			
	b) In the 3 weeks **before** you became ill did you **eat** any rodents?			
	c) In the 3 weeks **before** you became ill did you **touch** any rodents?			
	d) Have you noticed any rodents or rodents' urine or faeces in the house or in the immediate surroundings of the house or on food stuff?			
	e) Do you or your family dry food materials in the open, e.g. rice, garri, fish, utensils etc.			
	f) Do you or your family leave containers of food stuff uncovered?			
	g) What type of house or shelter do you live in, select as appropriate?			
	i. Mud house			
	ii. Brick house			

	iii. Temporary shelter (Camps)			
	iv. Hostel			
	v. Other			
	If others, then please describe			
5)	In the **last** 3 weeks **before** you became unwell, did you participate in any form of burial rites (carry, wash or touch corpse)?			
6)	Have you ever had a laboratory **positive** test for Lassa fever?			
7)	If yes, please state approximately when.			

Section 6: Specimen Collection and Results

26: Specimen collection done? ☐ Yes ☐ No. If yes, what samples? ☐ Blood ☐ Urine ☐ Buccal swab Other________________

**Lassa fever testing**	**Sample Collection date** (dd/mm/yyyy)	**Result release date** (dd/mm/yyyy)	**Result**
Lassa fever PCR			[ ] Positive<br>[ ] Negative<br>[ ] rejected sample

If result is negative, what other differential test was conducted?

1. None [ ]
2. Yellow fever [ ] Positive [ ] Negative
3. Others specify [ ] Positive [ ] Negative

Section 7: Outcome and Discharge details

27. Has a list of contacts been compiled for the case? [ ] Yes [ ] No

28. Did patient test positive for any other infection? [ ] Yes [ ] No If Yes, specify__________________________

29. If positive, date commenced with ribavirin: --------------------------------DD/MM/YYYY

30. **DISCHARGE DETAILS (this is needed to know the duration of treatment when it started and ended)**

Date of Discharge	________________________ **(dd/mm/yyyy)**
Outcome at discharge	Alive<br>1. Full recovery without sequelae at time of discharge [ ]<br>2. Full recovery with sequelae [ ]<br>• Hearing loss [ ]<br>• If pregnant, foetal loss [ ]<br>• Others specify: ____________________
	Dead [ ] Burial : Safe [ ] Unsafe [ ]
	Abscond [ ]

Form completed by: _________________________Designation________________

Date of Completion_________(dd/mm/yyyy) Signature: __________________________
