# Supplementary material for: Analysis of sociodemographic and clinical factors associated with Lassa fever disease and mortality in Nigeria
Source: PLOS Glob Public Health. 2022 Aug 26;2(8):e0000191. doi: 10.1371/journal.pgph.0000191 (PMC10022364; doi:10.1371/journal.pgph.0000191)
Supplement: S2 File — (PDF) [file pgph.0000191.s003.pdf]

NIGERIA CENTER FOR DISEASE CONTROL

NATIONAL LASSA FEVER CASE MANAGEMENT FORM

I. CASE IDENTIFICATION/ DEMOGRAPHIC DETAILS

Patient Name: **(Surname first)**	Name of Treatment Centre:	Residential Address **(Local Government Area inclusive):**<br><br>Patient Phone number(s):
**EPID ID:**		
☐ Male ☐ Female	Patient occupation<br>☐ Healthcare worker: ___________________________ (please specify)<br>☐ Non-Healthcare worker: _______________________ (please specify)	
Date of birth **(dd/mm/yyyy)**<br>____ /____ /___	Age: ________ ☐ Years ☐ Months	
Date of admission at facility: **(dd/mm/yyyy)**<br>___ /__ /___	Was patient transferred from another facility?<br>☐ Yes ☐ No ☐ Unknown. If yes, name of facility _____________________	

II. VITALS AT TRIAGE:

Heart rate/ Pulse rate (bpm):	Respiratory Rate (cpm):	Temperature (°C):
BP (mmHg): _____ (systolic) _____ (diastolic)	O_2 saturation room air (%): ____	Capillary refill > 3 sec? ☐ Yes ☐ No
Mental status:<br>Blantyre Score (Children)____________<br>Glasgow Coma Score (Adult)__________	Weight (kg): _____<br>Self-reported height (cm): _____	Mid-upper arm circumference (for patients less than 5 years of age) (cm)<br>_______

III. CLINICAL DETAILS (on admission)

Onset of first symptoms **(dd/mm/yyyy):** ____/___ /___<br><br>Date of admission at treatment centre **(dd/mm/yyyy):**<br>____/_____/______<br><br>Admitted to what type of bed? ☐ Ward ☐ ICU	If patient is female:<br>A. Is she pregnant? ☐ Yes ☐ No Pregnancy test result________<br><br>LMP **(dd/mm/yyyy):** ____/___ /____<br>EGA (weeks)________<br><br>Bilateral painful breast engorgement ☐ Yes ☐ No<br><br>B. Post-partum (up to 6 weeks)? ☐ Yes ☐ No<br>If yes, delivery date **(dd/mm/yyyy):** ____ /____/______<br><br>Mode of delivery: ☐ SVD ☐ Caesarean Section ☐ Assisted Delivery
If patient is a neonate:<br>A. Mother treated with ribavirin during pregnancy? ☐ Yes ☐ No<br>B. History of PROM? ☐ Yes ☐ No<br>C. Mode of delivery: ☐ SVD ☐ Caesarean Section ☐ Assisted Delivery<br>D. APGAR Score: _____ (1 minute), _______ (5 minute)<br>E. Weight at birth: _______kg<br>F. Feeding options employed ☐ Breastmilk ☐ Formula ☐ Mixed Feeding	
**Comorbid conditions**<br>Chronic heart failure (including congenital disease) ☐ Yes ☐ No ☐ Unknown<br>Chronic kidney disease ☐ Yes ☐ No ☐ Unknown<br>Chronic liver disease ☐ Yes ☐ No ☐ Unknown<br>Chronic neurologic condition (including cerebral palsy) ☐ Yes ☐ No ☐ Unknown<br>Chronic pulmonary disease ☐ Yes ☐ No ☐ Unknown<br>Diabetes Mellitus ☐ Yes ☐ No ☐ Unknown<br>Hepatitis ☐ Yes ☐ No ☐ Unknown	HIV ☐ Yes ☐ No ☐ Unknown<br>If yes, on ART? ☐ Yes ☐ No ☐ Unknown<br>Hypertension ☐ Yes ☐ No ☐ Unknown<br>Malignancy ☐ Yes ☐ No ☐ Unknown<br>If yes, on chemotherapy ☐ Yes ☐ No ☐ Unknown<br>Malnutrition ☐ Yes ☐ No ☐ Unknown<br>Sickle Cell Disease ☐ Yes ☐ No ☐ Unknown<br>Tuberculosis ☐ Yes ☐ No ☐ Unknown<br>Others (Pls specify) ________________________________

NIGERIA CENTER FOR DISEASE CONTROL

NATIONAL LASSA FEVER CASE MANAGEMENT FORM

Symptoms (at presentation)

Fever ☐ Yes ☐ No ☐ Unknown
Weakness ☐ Yes ☐ No ☐ Unknown
Malaise ☐ Yes ☐ No ☐ Unknown
Myalgia ☐ Yes ☐ No ☐ Unknown
Anorexia ☐ Yes ☐ No ☐ Unknown
Bleeding from orifice(s) ☐ Yes ☐ No ☐ Unknown
Sore throat ☐ Yes ☐ No ☐ Unknown

Headache ☐ Yes ☐ No ☐ Unknown
Chest pain ☐ Yes ☐ No ☐ Unknown
Cough ☐ Yes ☐ No ☐ Unknown
If yes, is cough productive of sputum? ☐ Yes ☐ No ☐ Unknown
Diarrhoea ☐ Yes ☐ No ☐ Unknown
Dark Coloured Urine ☐ Yes ☐ No ☐ Unknown
Hearing Impairment ☐ Yes ☐ No ☐ Unknown

Nausea ☐ Yes ☐ No ☐ Unknown
Vomiting ☐ Yes ☐ No ☐ Unknown
Irritability/ Confusion ☐ Yes ☐ No ☐ Unknown
Abdominal pain ☐ Yes ☐ No ☐ Unknown
Lethargy ☐ Yes ☐ No ☐ Unknown
Joint Pain ☐ Yes ☐ No ☐ Unknown
Others (Pls specify) ______________

Signs (at presentation)

Pharyngeal erythema ☐ Yes ☐ No ☐ Unknown
Pharyngeal exudate ☐ Yes ☐ No ☐ Unknown
Conjunctival injection/bleeding ☐ Yes ☐ No ☐ Unknown
Oedema of face/neck ☐ Yes ☐ No ☐ Unknown
Tender abdomen ☐ Yes ☐ No ☐ Unknown
Sunken eyes or fontanelle ☐ Yes ☐ No ☐ Unknown
Loss of skin turgor ☐ Yes ☐ No ☐ Unknown
Palpable liver ☐ Yes ☐ No ☐ Unknown
Palpable spleen ☐ Yes ☐ No ☐ Unknown
Rash ☐ Yes ☐ No ☐ Unknown
Jaundice ☐ Yes ☐ No ☐ Unknown

Enlarged lymph nodes ☐ Yes ☐ No ☐ Unknown
If yes, distribution________________________
Lower extremity oedema ☐ Yes ☐ No ☐ Unknown
Bleeding ☐ Yes ☐ No ☐ Unknown
If yes, site of bleeding:
Nose ☐ Yes ☐ No
Mouth ☐ Yes ☐ No
Vagina ☐ Yes ☐ No
Rectum ☐ Yes ☐ No
Sputum ☐ Yes ☐ No
Urine ☐ Yes ☐ No
Others (Pls specify) ________________________

IV. SPECIMEN COLLECTION AND RESULTS

Specimen collection? ☐ Yes ☐ No. If yes, what samples? ☐ Blood ☐ CSF ☐ Others______________

Lassa fever testing	Collection date (dd/mm/yyyy)	Result
Lassa PCR (admission)	_____/_____/______	☐ Pos ☐ indeterminate. Cycle time_______
Lassa PCR (Repeat 1)	_____/_____/______	☐ Pos ☐ Neg ☐ indeterminate. Cycle time_______
Lassa PCR (Repeat 2)	_____/_____/______	☐ Pos ☐ Neg ☐ indeterminate. Cycle time_______
Lassa PCR (Repeat 3)	_____/_____/______	☐ Pos ☐ Neg ☐ indeterminate. Cycle time_______
Malaria RDT	_____/_____/______	☐ Pos ☐ Neg ☐ indeterminate
Random Blood Glucose	_____/_____/______	_________________ (mmol/L)

Did patient test positive for any other infection? ☐ Yes ☐ No If Yes, pls specify_______________________________

Other clinical laboratory tests done on admission (ND = not done)

Urinalysis
Haemoglobinuria ☐ Pos ☐ Neg ☐ ND
Proteinuria ☐ Pos ☐ Neg ☐ ND
Haematuria ☐ Pos ☐ Neg ☐ ND

Blood gases (Arterial/venous)
pH_____, pCO_2_____, PaO_2 _____, HCO_3______.
Oxygen therapy at time of blood gas (L/min) ____________

Laboratory tests from admission. If not done, write ND (ND = not done).

	Admission	Discharge		Admission	Discharge
**Haematology Tests**					
Haemoglobin/ PCV (g/L or %)			RBC ($x10^{12}$/L)		
WBC count ($x10^9$/L)			PT		
Platelets (x 10^9/L)			aPTT (seconds)		
**Biochemistry Tests**					
Creatinine (umol/L)			Lactate (mmol/L)		
Potassium (mmol/L)			Creatinine Kinase/ EGFR (mmol/L)		
Sodium (mmol/L)			ALT/SGPT (U/L)		
Urea (mmol/L)			AST/SGOT (U/L)		
Creatinine kinase (U/L)			Total bilirubin (umol/L)		

Modified from WHO VHF/SARI Case_Record_Form 2016.

NATIONAL LASSA FEVER CASE MANAGEMENT FORM

Calcium (mmol/L)			Conj. bilirubin (umol/L)		

V. Complications at any time (OD= onset date, format dd/mm/yyyy)

Acute Kidney Injury ☐ Yes ☐ No ☐ Unknown OD___/____/__	Hyperglycaemia ☐ Yes ☐ No ☐ Unknown OD ___/___ /___
Anaemia ☐ Yes ☐ No ☐ Unknown OD___/____/_____	Hypoglycaemia ☐ Yes ☐ No ☐ Unknown OD ___/___ /___
Bacteraemia ☐ Yes ☐ No ☐ Unknown OD___/___ /___	Meningitis* ☐ Yes ☐ No ☐ Unknown OD___/____/_____
Bleeding ☐ Yes ☐ No ☐ Unknown OD___/____/_____	Seizure ☐ Yes ☐ No ☐ Unknown OD___/____/____
Coma (GCS < 8) ☐ Yes ☐ No ☐ Unknown OD ___/___ /____	Shock ☐ Yes ☐ No ☐ Unknown OD___/____/_____
Confusion ☐ Yes ☐ No ☐ Unknown OD___/____/_____	Others (Pls specify) ____________________________________ OD___/___ /____
Hearing impairment ☐ Yes ☐ No ☐ Unknown OD___/____/_____	

***meningitis defined either clinically or with result of LP M/C/S**

VI. TREATMENT INFORMATION: (please include loading dose, maintenance and switch to oral therapy)

Did patient receive ANY antimicrobial/antiviral therapy? ☐ Yes ☐ No

**Type**	**Dose**	**Route**	**Frequency**	**Start date (dd/mm/yyyy)**	**End date (dd/mm/yyyy)**
Ribavirin		☐ **IV** ☐**oral**	☐ **once** ☐**Q6H** ☐ **Q8H**		
		☐ **IV** ☐**oral**	☐ **once** ☐**Q6H** ☐ **Q8H**		
		☐ **IV** ☐**oral**	☐ **once** ☐**Q6H** ☐ **Q8H**		
		☐ **IV** ☐**oral**	☐ **once** ☐**Q6H** ☐ **Q8H**		
		☐ **IV** ☐**oral**	☐ **once** ☐**Q6H** ☐ **Q8H**		
Antibacterial (Pls specify)		☐ **IV** ☐**oral** **Other routes (pls specify)** ____________			
Antimalarial (Pls specify) ________________		☐ **IV** ☐**oral**			
Others (Pls specify)		☐ **IV** ☐**oral** **Other routes (pls specify)**			

Did the patient receive any of the following during hospitalization?

Other Specialist Review

1.____________________________________ Indication(s): ____
2.____________________________________ Indication(s): ____
3.____________________________________ Indication(s): ____
4.____________________________________ Indication(s): ____
5.____________________________________ Indication(s): ____

Oral rehydration salts ☐ Yes ☐ No	Oxygen therapy ☐ Yes ☐ No	IV fluid therapy ☐ Yes ☐ No
Vasopressors/inotropes. ☐ Yes ☐ No	Mechanical ventilation ☐ Yes ☐ No	Access route: ☐ PIV ☐ CVC ☐ Intra-osseous

Blood transfusion. ☐ Yes ☐ No<br>If Yes, number of pints______________	Renal replacement therapy ☐ Yes ☐ No<br>If Yes, number of dialysis sessions____________________

VII. DISCHARGE DETAILS

Date of Discharge/transfer from treatment centre/death	____ /_______/_________ **(dd/mm/yyyy)**
Final Diagnosis: ☐ Lassa fever ☐ Others (Pls specify)<br>**Outcome at discharge**	

Modified from WHO VHF/SARI Case_Record_Form 2016.

NATIONAL LASSA FEVER CASE MANAGEMENT FORM

☐ Full recovery WITHOUT sequelae at time of discharge
☐ Full recovery WITH sequelae (Tick appropriate box) ☐ hearing loss ☐ if pregnant, spontaneous abortion ☐ others: (Pls specify)____
☐ Death of patient
☐ Referral to another facility. (Please specify name of facility) ____
☐ Discharge against medical advice

Form completed by: _____________________________________ **Date of Completion(dd/mm/yyyy) ____________________**
